# Quantitative ultrasound image assessment of the optic nerve subarachnoid space during 90-day head-down tilt bed rest

**DOI:** 10.1038/s41526-024-00347-x

**Published:** 2024-01-17

**Authors:** Yuan Xie, Yingdi Fu, Yaqi Shao, Lina Qu, Jiangang Yang, Chengjia Yang, Kun Zhou, Kai Li, Zi Xu, Dong Xu, Kai Cao, Ning Tian, Ke Lv, Linjie Wang, Yaping Wang, Ningli Wang, Yinghui Li

**Affiliations:** 1 Beijing Tongren Eye Center, Beijing Tongren Hospital, Capital Medical University, Beijing Ophthalmology and Visual Sciences Key Laboratory, Beijing, 100730 China; 2 Beijing Institute of Ophthalmology, Beijing Tongren Hospital, Capital Medical University, Beijing, 100005 China; 3 China Astronaut Research and Training Center, State Key Lab of Space Medicine Fundamentals and Application, No. 26 Beiqing Road, Haidian District, Beijing, 100094 China; 4 Xi’an No.1 Hospital; Shanxi Institute of Ophthalmology; Shanxi Key Laboratory of Ophthalmology; Clinical Research Center for Ophthalmology Diseases of Shanxi Province; the First Affiliated Hospital of Northwestern University, Xi’an, 710002 Shanxi Province China

**Keywords:** Optic nerve diseases, Diagnostic markers

## Abstract

The elevation in the optic nerve sheath (ONS) pressure (ONSP) due to microgravity-induced headward fluid shift is the primary hypothesized contributor to SANS. This longitudinal study aims to quantify the axial plane of the optic nerve subarachnoid space area (ONSSA), which is filled with cerebrospinal fluid (CSF) and expands with elevated ONSP during and after head-down tilt (HDT) bed rest (BR). 36 healthy male volunteers (72 eyes) underwent a 90-day strict 6° HDT BR. Without obtaining the pre-HDT data, measurements were performed on days 30, 60, and 90 during HDT and at 6 recovery time points extended to 180-days (R + 180) in a supine position. Portable B-scan ultrasound was performed using the 12 MHz linear array probe binocularly. The measurements of the ONS and the calculation of the ONSSA were performed with ImageJ 1.51 analysis software by two experienced observers in a masked manner. Compared to R + 180, the ONSSA on HDT30, HDT60, and HDT90 exhibited a consistently significant distention of 0.44 mm^2^ (95% CI: 0.13 to 0.76 mm^2^, *P* = 0.001), 0.45 mm^2^ (95% CI: 0.15 to 0.75 mm^2^, *P* = 0.001), and 0.46 mm^2^ (95% CI: 0.15 to 0.76 mm^2^, *P* < 0.001), respectively, and recovered immediately after HDT on R + 2. Such small changes in the ONSSA were below the lateral resolution limit of ultrasound (0.4 mm) and may not be clinically relevant, possibly due to ONS hysteresis causing persistent ONS distension. Future research can explore advanced quantitative portable ultrasound-based techniques and establish comparisons containing the pre-HDT measurements to deepen our understanding of SANS.

## Introduction

Following long-duration spaceflight, astronauts would suffer from a range of neuro-ophthalmic changes collectively named spaceflight-associated neuro-ocular syndrome (SANS)^1^. SANS is characterized by decreased near-visual acuity, global flattening, optic disc edema, and choroidal folds^2^. It has the potential to induce permanent ophthalmic changes during long-duration spaceflight missions^3,4^. Therefore, understanding the pathophysiology of SANS is currently a major priority in space medicine research. It is hypothesized that the development of SANS is related to the elevations in intracranial pressure (ICP) due to microgravity-induced cephalad fluids shift^5^ and also related to spaceflight-induced compartmentalization of cerebrospinal fluid in the optic nerve subarachnoid space with locally elevated ONSP^6^. However, direct ICP monitoring methods are invasive and complicated, making them impractical for routine application during spaceflight missions. The optic nerve is surrounded by the optic nerve sheath (ONS), including the pia, arachnoid, and dura mater layers. The optic nerve subarachnoid space (ONSS) is formed between the arachnoid layer and the pia layer, and it is filled with CSF, which expands with elevated ICP or ONSP^7–9^.

The ONS diameter (ONSD)^10^ at 3 mm posterior to the globe was assessed in several crew members after a 6-month mission using MRI^11^ and transorbital ultrasound, showing inconsistent results^10^. Several methodological discrepancies may have contributed to this inconsistent ONSD assessment results, including the differences in transducer frequency^10,12,13^, MI^10,12,13^, ONSD delineation^14–18^, and anatomical landmarks for calculating ONSD depth^12^. Also, there were challenges in assessing ONSD measurement quality without a sample image, potential issues like researcher experiences or blinding during measurements, and the distinction between the inclusion of the dura in ONSD measurement and the measurement at the interface between the SAS and dura^19^. Furthermore, the collagen fibers of the ONS were primarily oriented axially and helically but with inhomogeneity^9^, and the CSF shear stress distribution across the optic nerve sheath wall may also show inhomogeneity^20^. Our previous neurological study has demonstrated the correlation coefficient between the ICP and ONSSA was the highest compared with that of the ICP and ONSD or optic nerve subarachnoid space width (ONSSW) at 3 mm point using non-portable ultrasonography^21^. Therefore, this study considered that measuring the optic nerve subarachnoid space area (ONSSA) in the axial plane could serve as a surrogate for quantifying the volume of the optic nerve subarachnoid space and a more robust indicator of ONSP compared to measuring the width at a single 3 mm point. The changes that occurred in the ONSSA assessed by portable ultrasonography during the microgravity challenge were still unknown.

Long-duration head-down tilt (HDT) bed rest (BR) is internationally recognized as a suitable approach for examining microgravity-caused physiologic effects of spaceflight and testing countermeasures in a ground-based model^22^. The present study aims to develop a reliable ultrasound-based measurement method to investigate the effects of microgravity on surrogate measurements of ONSP.

## Results

### Anthropometric and hemodynamic characteristics of participants

This longitudinal study included 72 eyes from 36 Chinese male volunteers, who had a mean (SD) age of 32.4 (5) years and a mean (SD) body mass index (BMI) of 22.20 (2.12) kg/m^2^ before HDT. All the subjects completed a strict 90-day HDT BR. Because the pre-HDT ONS data were not collected, the baseline level was established using the R + 180 data. Repeated-measures ANOVA was applied to analyze the differences in BMI, mean blood pressure (MBP), heart rate, head circumference, waist circumference, and hip circumference across different exam time points. Bonferroni correction was applied for multiple comparisons. The results indicates that the hip circumference decreased during HDT BR, with changes on HDT30, HDT60, and HDT 90 of -3.97 cm (95% confidence interval [CI]: -6.14 to -1.8 cm, *P* < .001), -2.97 cm (95% CI: -5.37 to -0.57 cm, *P* = .005), and -2.36 cm (95% CI: -4.14 to -0.58 cm, *P* = .002) compared to the value on R + 180, respectively. The head circumference significantly increased on HDT30 (1.15 cm, 95% CI: 0.25 to 2.05 cm, *P* = .004) and HDT90 (0.89 cm, 95% CI: 0.15 to 1.63 cm, *P* = 0.008) compared to the value on R + 180. The BMI and the waist circumference remained stable throughout the entire HDT period (all *P* > .05). The MBP was higher on HDT30 (8.76 mmHg, 95% CI: 2.83 to 14.68 mmHg, *P* = 0.001), HDT60 (7.22 mmHg, 95% CI: -0.77 to 15.22 mmHg, *P* = 0.109), and HDT90 (7.56 mmHg, 95% CI: 0.17 to 14.94 mmHg, *P* = 0.042) compared with the value on R + 180. The heart rate significantly decreased during HDT and returned to R + 180 level on R + 90 (Table 1).

**Table 1:** Statistics (mean ± SD) of anthropometric and hemodynamic characteristics of participants (*n* = 36) during head down Tilt(HDT) bed best(BR).

	Pre	HDT30d	HDT60d	HDT90d	R + 7d	R + 14d	R + 30d	R + 90d	R + 180d
BMI, kg/m^2^	22.2 ± 2.12	21.47 ± 0.35	21.46 ± 0.35	21.29 ± 0.34	–	–	22.36 ± 0.34	21.79 ± 0.35	21.87 ± 0.35
Head circumference, cm	**–**	**55.89** ± **1.96**	**–**	**55.63** ± **2.15**	55.24 ± 1.65	54.97 ± 1.82	54.67 ± 1.74	54.99 ± 1.91	54.74 ± 1.85
Waist circumference, cm	–	75.01 ± 6.74	74.89 ± 5.81	74.82 ± 6.5	74.82 ± 6.91	75.86 ± 6.86	74.44 ± 5.99	73.65 ± 5.88	76.06 ± 5.93
Hipline, cm	**–**	**87.97** ± **5.85**	**88.97** ± **4.62**	**89.58** ± **3.7**	90.71 ± 4.0	91.06 ± 3.99	90.49 ± 3.82	88.26 ± 3.88	91.94 ± 3.88
MBP, mmHg	86.36 ± 9.14	**89.39** ± **1.1**	**87.98** ± **1.25**	**88.55** ± **1.09**	86.89 ± 1.09	83.98 ± 0.84	82.02 ± 0.87	84.08 ± 1.04	81.29 ± 1.07
Heart Rate, bpm	77.78 ± 10.04	**62.5** ± **13.22**	**63.81** ± **8.5**	**62.69** ± **8.38**	**66.94** ± **9.78**	**65.53** ± **10.19**	**64.08** ± **8.07**	73.06 ± 9.51	73.11 ± 9.79

The bold one indicated significantly difference compared with 180-day at recovery time(*p* < 0.05) (ANOVA).

Body Mass Index =weight/height^2^, MBP(Mean Blood Pressure)=1/3×systolic blood pressure+2/3×diastolic blood pressure.

### Optic nerve sheath measurements

**Fig. 1:**
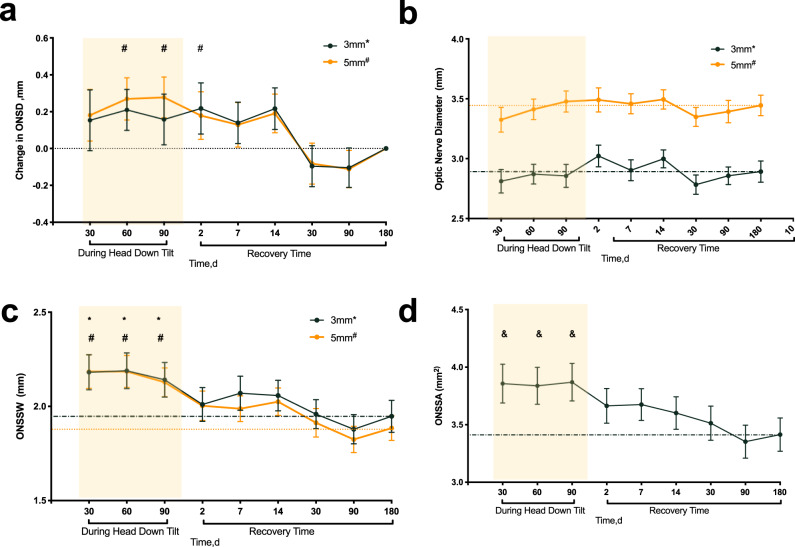
Optic Nerve sheath complex variation in volunteers during 30, 60, 90 days of the head down tilt bed rest and 2, 7, 14, 30, 90, 180 days of recovery. Error bars represent the 95% confidence interval of the mean. The “*” and “#” represent ONSD (**a**), OND (**b**), ONSSW (**c**) at 3 mm and 5 mm posterior to the optic disc, respectively, which significantly increased in comparison to the values on day 180 of the recovery time (R + 180). The “&” indicates ONSSA (**d**) between 3 to 5 mm posterior to the optic disc significantly increased compared with that on R + 180. ONSD optic nerve sheath diameter, OND optic nerve diameter, ONSSW the width of the optic nerve subarachnoid space, ONSSA the transversal area of the optic nerve subarachnoid space.

The quantification of ultrasound ONSD during the 90-day HDT is presented in Table 2. The intraclass correlation coefficient (ICC) was used to assess the inter- and intra-observer reliability, and demonstrated excellent reliability (Table 3). A mixed linear model was used to compare repeated measurements of ONS parameters at each time point to adjust for the correlation between the two eyes of each subject. The ONSD at 3 mm posterior to the optic disc during the HDT was nonsignificantly elevated compared to the R + 180 level, with average distensions of 0.15 mm (95% CI: -0.09 to 0.4 mm, *P* = 0.682), 0.21 mm (95% CI: -0.01 to 0.43 mm, *P* = 0.063), and 0.17 mm (95% CI: -0.073 to 0.4 mm, *P* = 0.44) on HDT30, HDT60, and HDT90, respectively. The ONSD at 5 mm during the HDT was significantly larger than that on R + 180 and returned to the R + 180 level on R + 2, with average distensions of 0.18 mm (95% CI: -0.043 to 0.405 mm, *P* = .214), 0.29 mm (95% CI: 0.065 to 0.474 mm, *P* = 0.003), and 0.28 mm (95% CI: 0.062 to 0.493 mm, *P* = 0.004) on HDT30, HDT60, and HDT90, respectively (Fig. 1a). The optic nerve diameter (OND) at 3 mm and 5 mm posterior to the optic disc were consistent throughout the entire HDT period (*P* > 0.99) (Fig. 1b).

**Table 2:** Measurements of Optic nerve sheath diameter, optic nerve diameter, optic nerve subarachnoid space width and area during HDT90d and at recovery time points.

	HDT30d	HDT60d	HDT90d	R + 2d	R + 7d	R + 14d	R + 30d	R + 90d	R + 180d	*P* value ^a^
ONSD 3,mm	4.99 ± 0.55	5.04 ± 0.44	5 ± 0.53	5.06 ± 0.55	4.98 ± 0.47	5.06 ± 0.37	4.74 ± 0.44	4.74 ± 0.45	4.84 ± 0.51	<0.001
ONSD 5, mm	5.51 ± 0.5	**5.6** ± **0.42**	**5.61** ± **0.47**	**5.51** ± **0.54**	5.46 ± 0.45	5.52 ± 0.42	5.25 ± 0.43	5.22 ± 0.45	5.33 ± 0.46	<0.001
Average	4.99 ± 0.55	5.05 ± 0.45	5 ± 0.53	5.06 ± 0.55	4.98 ± 0.47	5.06 ± 0.37	4.74 ± 0.44	4.74 ± 0.45	4.84 ± 0.51	<0.001
*P* value^**b**^	<0.001	<0.001	<0.001	<0.001	<0.001	<0.001	<0.001	<0.001	<0.001	
OND 3,mm	2.81 ± 0.42	2.87 ± 0.34	2.86 ± 0.41	3.02 ± 0.38	2.9 ± 0.37	3 ± 0.32	2.82 ± 0.47	2.86 ± 0.31	2.89 ± 0.38	0.017
OND 5,mm	3.33 ± 0.44	3.41 ± 0.37	3.48 ± 0.38	3.49 ± 0.43	3.46 ± 0.36	3.49 ± 0.34	3.35 ± 0.34	3.39 ± 0.4	3.44 ± 0.37	0.009
Average	2.81 ± 0.42	2.87 ± 0.35	2.86 ± 0.41	3.02 ± 0.38	2.9 ± 0.37	3 ± 0.32	2.82 ± 0.47	2.86 ± 0.31	2.89 ± 0.38	<0.001
*P* value^b^	<0.001	<0.001	<0.001	<0.001	<0.001	<0.001	<0.001	<0.001	<0.001	
ONSSW 3,mm	**2.19** ± **0.4**	**2.22** ± **0.4**	**2.15** ± **0.38**	2.03 ± 0.4	2.08 ± 0.38	2.06 ± 0.35	1.92 ± 0.42	1.88 ± 0.33	1.95 ± 0.36	<0.001
ONSSW 5,mm	**2.19** ± **0.38**	**2.19** ± **0.36**	**2.13** ± **0.33**	2.02 ± 0.36	2 ± 0.31	2.03 ± 0.31	1.9 ± 0.35	1.82 ± 0.3	1.88 ± 0.28	<0.001
Average	2.18 ± 0.4	2.19 ± 0.41	2.14 ± 0.39	2.01 ± 0.38	2.07 ± 0.38	2.06 ± 0.35	1.96 ± 0.33	1.88 ± 0.33	2.02 ± 0.35	<0.001
*P* value^b^	0.929	0.983	0.717	0.871	0.079	0.479	0.177	0.172	0.07	
ONSSA 3-5,mm^2^	**3.86** ± **0.72**	**3.84** ± **0.67**	**3.87** ± **0.69**	3.66 ± 0.64	3.68 ± 0.59	3.6 ± 0.6	3.51 ± 0.63	3.35 ± 0.61	3.41 ± 0.61	<0.001

*P* value ^a^ : significantly difference between multiple time points(Mixed linear model).

*P* value^b^: statistical significance of the difference between the positions at 3 and 5 mm within the same group (ANOVA).

The bold one indicated significantly difference compared with 180-day at recovery time(*p* < 0.05).

ONSD 3/5 = optic nerve sheath diameter at 3 mm or 5 mm behind the optic disc.

OND 3/5 = optic nerve diameter at 3 mm or 5 mm behind the optic disc.

ONSSW 3/5 = the width of the optic subarachnoid space at 3 mm or 5 mm behind the optic disc.

ONSSA 3–5 = the area of the optic subarachnoid space from 3 mm to 5 mm behind the optic disc.

*HDT* head down tilt bed rest time, *R* recovery time.

**Table 3:** Inter and Intra-observer Reproducibility of Optic Nerve Diameter, Optic Nerve Sheath Diameter and Area of the Optic Nerve Subarachnoid Space Measurements.

Measurements	Interobserver Difference (95%LoA),mm	ICC (95%CI)	Intra-observer Difference (95%LoA),mm	ICC (95%CI)
ONSD 3, mm	−0.02 (−0.06,-0.01)	0.87 (0.82,0.91)	0.01 (−0.02,0.04)	0.96 (0.94,0.97)
ONSD 5, mm	−0.11 (−0.05,-0.02)	0.72 (0.61,0.8)	0.01 (−0.03,0.05)	0.91 (0.87,0.94)
OND 3, mm	−0.02 (−0.07,0.05)	0.85 (0.79,0.89)	0.01 (−0.03,0.3)	0.91 (0.87,0.94)
OND 5, mm	−0.04 (−0.07,-0.01)	0.88 (0.83,0.92)	−0.02 (−0.07,0.04)	0.81 (0.73,0.87)
ONSSA 3–5, mm^2^	−0.07 (−0.12,-0.02)	0.86 (0.79,0.90)	0.03 (0.00,0.06)	0.95 (0.92,0.96)

ONSD 3/5 = optic nerve sheath diameter at 3 mm or 5 mm behind the optic disc.

OND 3/5 = optic nerve diameter at 3 mm or 5 mm behind the optic disc.

ONSSA 3–5 = the area of the optic subarachnoid space from 3 mm to 5 mm behind the optic disc.

*LoA* limits of agreement, *ICC* intraclass correlation coefficient.

The ONSSW at both 3 mm and 5 mm posterior to the optic disc were significantly distended. The average changes in ONSSW at 3 mm from HDT30, HDT60, and HDT90 to R + 180 were 0.25 mm (95% CI: 0.07 to 0.421 mm, *P* = 0.001), 0.27 mm (95% CI: 0.092 to 0.447 mm, *P* < 0.001), and 0.21 mm (95% CI: 0.35 to 0.379 mm, *P* = 0.009), respectively. The average changes in the ONSSW at 5 mm from HDT30, HDT60, and HDT90 to R + 180 were 0.3 mm (95% CI: 0.16 to 0.455 mm, *P* < 0.001), 0.31 mm (95% CI: 0.16 to 0.459 mm, *P* < 0.001), and 0.24 mm (95% CI: 0.104 to 0.384 mm, *P* < 0.001), respectively. The ONSSW recovered to the R + 180 level on R + 2 (Fig. 1c).

**Fig. 2:**
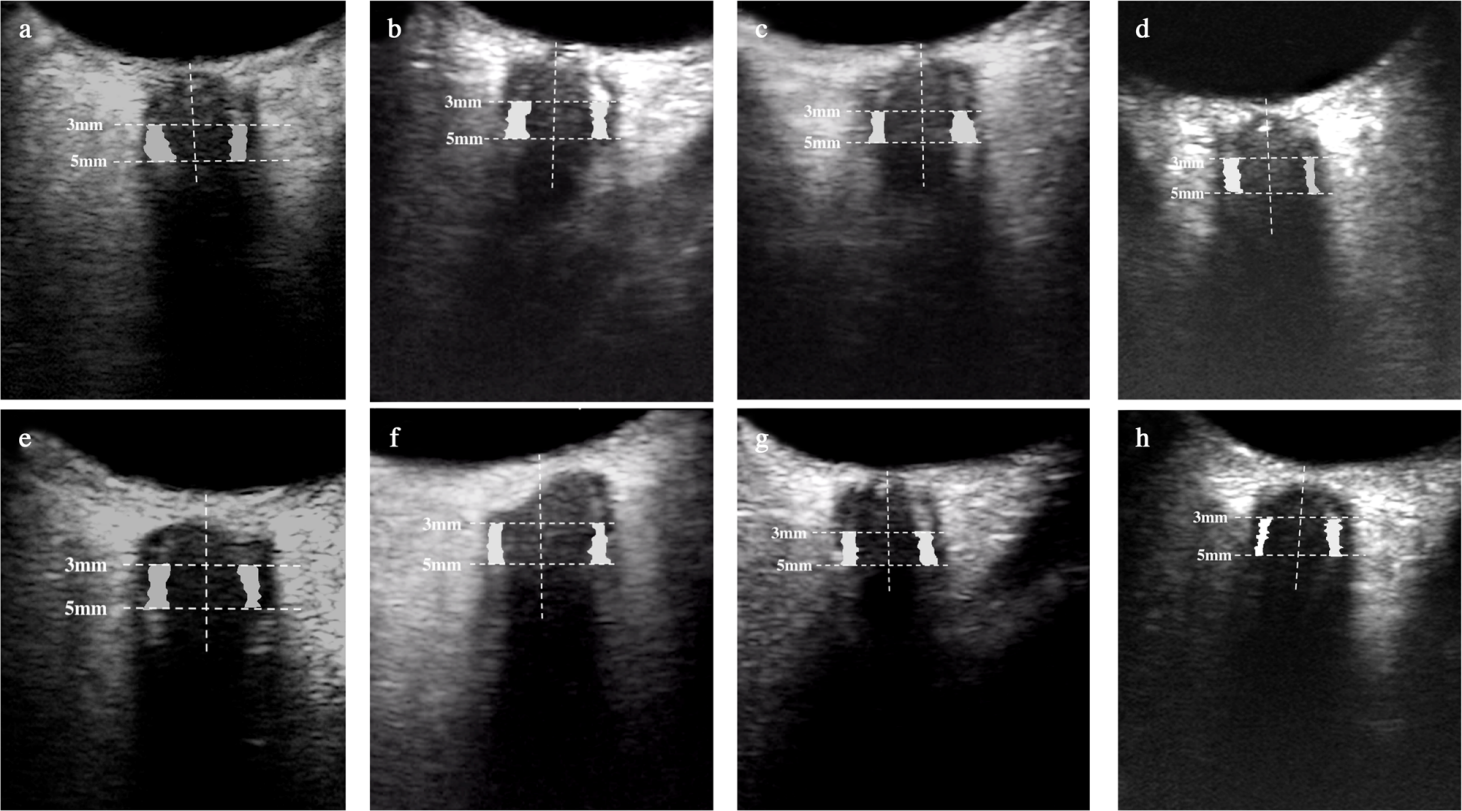
Representative images of optic nerve subarachnoid space area between 3 and 5 mm posterior to the optic disc (white shaded area) variations during the 90-day head down tilt bed rest and on recovery day 180. ONSSA distensions were showed on HDT30d (**a**, **e**), HDT60d (**b**, **f**), HDT90d (**c**, **g**), compared with R + 180d (**d**, **h**). The top and bottom lines indicated ONSSA in the right and left eye, respectively. HDT head down tilt bed rest, R recovery time.

The ONSSA between 3 mm to 5 mm posterior to the optic disc exhibited significant distention in comparison to R + 180, with average distensions of 0.44 mm^2^ (95% CI: 0.13 to 0.76 mm^2^, *P* = 0.001), 0.45 mm^2^ (95% CI: 0.15 to 0.75 mm^2^, *P* = 0.001), and 0.46 mm^2^ (95% CI: 0.15 to 0.76 mm^2^, *P* < 0.001) on HDT30, HDT60 and HDT90, respectively. The ONSSA recovered to the R + 180 level immediately after HDT on R + 2 (Fig. 1d). In Fig. 2, the ONSSA is highlighted as a white shaded area. The original, unmarked image can be found in Supplementary Figure 1.

The univariate linear regression demonstrated that the change in the ONSSA was associated with the change in MBP (Beta = 0.092, 95% CI: 0.016 to 0.169, *P* = 0.019), with no relations to BMI, head circumference, waist circumference, hip circumference, and heart rate (Table 4).

**Table 4:** Linear analysis regression on the association between systemic parameters and the area of the optic nerve subarachnoid space from 3 to 5 mm behind the optic disc.

Parameters	Univariate Linear Correlation	
	Beta(95 CI%)	*P* value
Body Mass Index, per 1 kg/m^2^ decrease	−0.054 (−0.136 to 0.028)	0.194
Mean Blood Pressure, per 10 mmHg	0.092 (0.016 to 0.169)	0.019
Heart Rate, per 10 bpm decrease	−0.048 (−0.203 to -0.006)	0.079
Hipline, per 1 cm decrease	0.017 (−0.043 to 0.008)	0.185
Waistline, per 1 cm decrease	−0.011 (0.034 to 0.012)	0.335
Head Circumference, per 1 cm	−0.007 (0.079 to 0.066)	0.856

## Discussion

The ultrasound-based quantification of intraorbital anatomy, which overcame some current limitations of ONSD measurements in SANS-related research^19^, revealed that microgravity has led to consistent ONS distension during the 90-day HDT in the current prospective longitudinal study. The results further confirmed that microgravity-induced maladaptive remodeling of the ONS may be attributed to the elevated ONSP. Also, assessing ONSSA using portable transorbital ultrasound could serve as a noninvasive method for evaluating SANS during spaceflight.

Histologic studies have indicated that the bulbous portion of the ONS is the thinnest area and appears to be the most distensible area when ICP is created in cadavers^7,23^. The ONSD at 3 mm posterior to the globe has been widely used in previous studies as the reference point to study the correlation with ICP^24^. Although both intra-reliability and inter-reliability of ONSD are good among individuals, the results vary across studies^25^. The discrepancies in methodological aspects that may contribute to the diversity in ONSD assessment should be considered^12,19^.

The transducer frequency and MI difference willaffect image characters. The use of a linear transducer with 12 MHz frequency in this study has provided the opportunity for more detailed measurements of both the optic nerve and the optic subarachnoid space. To ensure the safety of transorbital ultrasound measurements, this study adopted the “As Low As Reasonably Achievable” (ALARA) principle and the FDA-recommended MI index, which was lower than 0.23^26^. Several previous studies that applied lower-frequency probes (with 7.5 MHz being the most used frequency)^12^ reported images that displayed no striped bands in the dark region between the retrobulbar fat, which is different from measuring the optic subarachnoid space^10,13^. The present advanced approach using a high-frequency linear probe enabled the images to depict two hyperechoic striped bands within the hypoechoic region between the retrobulbar fat and also make the actual outlines of the optic nerve sheath more clearly visible. Therefore, the ONSSA assessment is possibly less susceptible to edge artifacts, thereby improving its power to predict ONSP.

In addition, the discrepancy in ONSD delineation may correspond to the inconsistent results in previous studies. Although there is no ambiguity that the optic nerve is represented by the hypoechoic longitudinal structure, there seems to be less agreement regarding the appearance of the pia mater, the subarachnoid space, and the dura mater. While some studies associate hyperechoic striped bands with this space, others suggest that the outer hypoechoic band represents the subarachnoid region. Microscopic studies have revealed that the subarachnoid space of the optic nerve is traversed by a meshwork of arachnoid trabeculae, with the highest density in the bulbar segment^7^. These septae consist of multiple acoustic interfaces, giving the subarachnoid space a partially hyperechoic appearance^16^. In a cadaver study by Steinborn, fluid injection led to the expansion of the hypoechoic optic nerve sheath, with the maximum dilatation appearing in the anterior part, 2 ~ 3 mm behind the optic nerve papilla. The meshwork of arachnoid trabeculae might be destroyed in the cadaver eye due to the injection of saline into the subarachnoid space interspace, manifesting as a hypoechoic area^27^. Therefore, most previous studies tend to consider the two stripped hyperechoic bands as representing the subarachnoid space^16,17,28,29^. There are two main measurement methods: ONSDint, where ONSD is measured at the border between the stripped hyperechoic bands and outer hypoechoic bands (S1), and ONSDext, where ONSD is measured outside the outer hypoechoic bands (D1). ONSDint exhibited a higher effect size between elevated ICP and normal controls (1.5 mm difference) when compared to the ONSDext (0.9 mm difference)^12^. Two smaller studies showed a higher diagnostic accuracy, as determined by the area under the curve (AUC) of receiver operating characteristics curves, for the detection of elevated ICP using ONSDext compared to ONSDint^14,30^. Since the inclusion of dura mater in ONSD measurements would lead to the overestimation of ONSD, ONSD measurement should start at the outer edge of the subarachnoid space (ONSDint)^31^. While most studies do not explicitly specify the structures considered in measurements, when details are provided, ONSDint was utilized more frequently (8.7%) than ONSDext (3.6%)^32^.

**Fig. 3:**
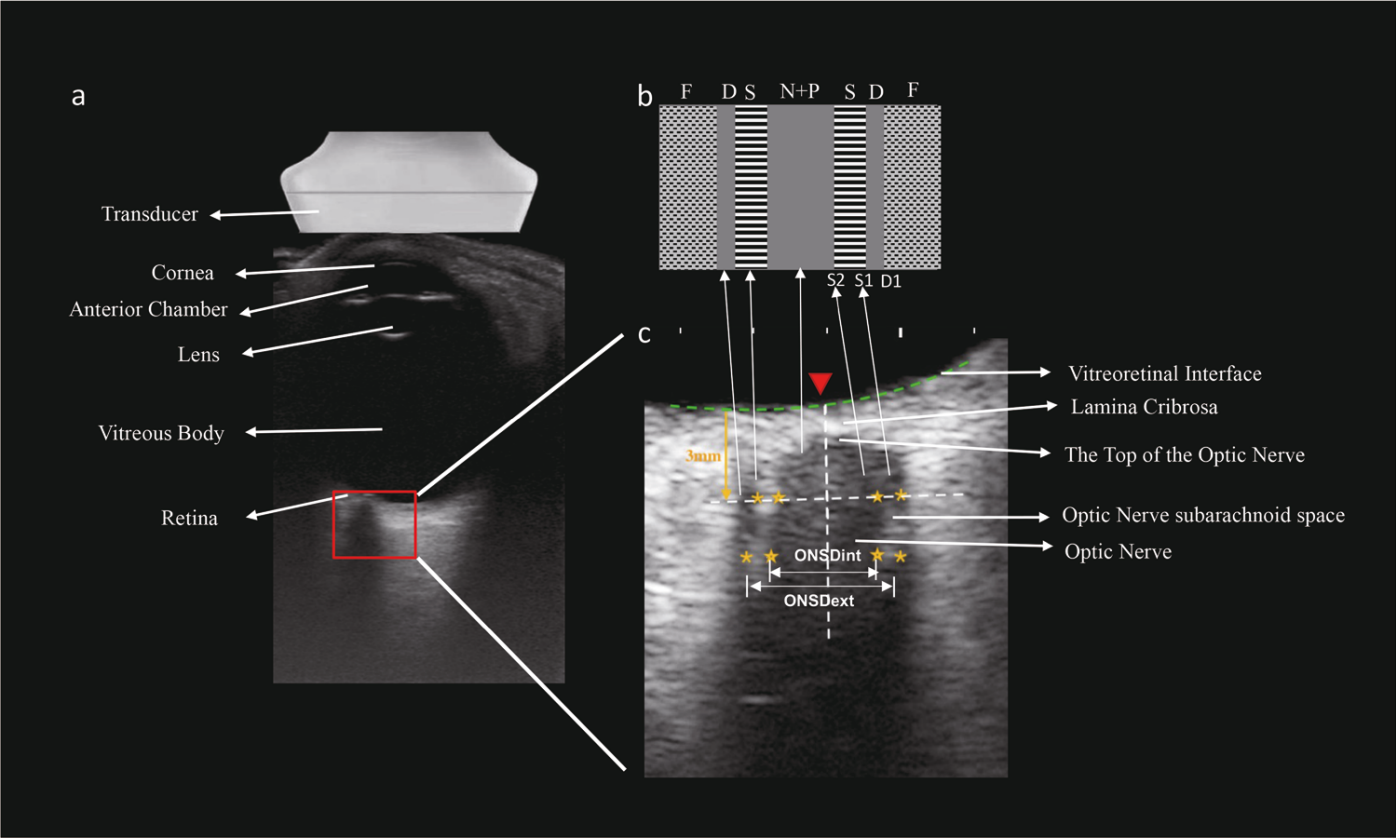
Optic nerve sheath complex measurements. **a** The 12 MHz transducer was placed over the upper eyelid of the test eye in a transversal plane. A sonographic slice provides the transversal view of the eye and the optic nerve sheath complex shows as a hypoechoic strip in the echogenic retrobulbar fat. **b** Schematic representation of the ultrasound optic nerve sheath complex. **c** The optic nerve sheath was measured at a depth of 3 and 5 mm posterior to the optic disc in the zoom mode. The depth marker was located at the vitreoretinal interface of the optic disc (Red inverted triangle), which was defined as 3 and 5 mm posterior to the globe. As shown in **b**, the pia mater (P) appeared as a dark structure fused with the optic nerve (N), the subarachnoid space (S) was the two hyperechoic striped bands, and the dura mater (D) showed as a dark line in-between the white retrobulbar fat and the hyperechoic striped bands. The optic nerve diameter (OND) was marked by the hyperechoic inner boundary on both sides (S1) and optic nerve sheath diameter (ONSD) was marked by the hyperechoic outer boundary on both sides (ONSDint, S2). The entire coronary transversal area of the optic nerve subarachnoid space (ONSSA) between 3 and 5 mm was outlined in the two hyperechoic striped bands (Yellow stars). D1 represented the external edge of the dura matter and ONSD (ONSDext).

Moreover, the anatomical landmarks used to calculate ONSD depth also varied among studies, resulting in different depth marker positions^12^. Three different anatomical landmark positions that have been reported are the vitreoretinal interface of the optic disc (Red inverted triangle in Fig. 3c), the lamina cribrosa, and the top of the optic nerve. The vitreoretinal interface of the optic disc was found to be clearer than the other two markers and was thus defined as the depth landmark. At the 7 mm measurement point posterior to the optic disc, the ONSSA edges were hard to identify in some subjects. Thus, the ONSSA was calculated perpendicular to the vertical axis of the scanning region, 3 ~ 5 mm behind the optic disc.

The ONSS is filled with CSF and distends with elevated ONSP, especially in the retrobulbar compartment. However, several factors can lead to irregular deformation of ONSS volume. The structure of ONSS is complex, with tiny arachnoid trabeculae and a septum separating it instead of a homogeneous space filled with CSF. Such a multichambered tubular system is sparse anteriorly and denser posteriorly, ending blindly in the brain, with considerable structural variations based on the location along the optic nerve^33^. The stretching force of ONSP applies pressure across the surface in a uniform direction, while shearing forces caused by the pressure wave of fluid can apply pressure in disparate directions that lead to the “fraying” of the fibers at breakpoints, resulting in increased porosity^20^. Furthermore, second harmonic generation microscopy has revealed the helical and axial orientation of the ONS collagen fibers. Variations in the collagen microstructure were also observed at different locations of the ONS wall, which could lead to inhomogeneities in stress distribution or residual stresses across the wall^9,34^. Combining the facts above and the fact that the optic nerve travels sinuously in both horizontal and vertical planes, measuring the ONSD at a specific cross-sectional position may not be sufficiently accurate. To increase precision, Chen et al. performed ONSD 3 mm measurements in both the transverse and vertical sections and calculated the average value^35^. Rohr et al. applied ONS cross area on sagittal MRI cuts^36^. The majority of previous studies have employed a transverse plane, and the image quality of transverse and sagittal planes does not differ or affect the measurement of ONSD^12^. The circumferential and axial stress-strain responses were found to be similar^9^. In this study, measuring ONSSA in the transverse plane could better counter errors caused by deformation and provide an accurate and convenient method for monitoring changes in ONSP^30^. Our previous study has demonstrated that ICP was more closely related to ONSSA between 3 and 7 mm than to ONSD at the 3 mm point, as assessed by the non-portable ultrasonography in the neurological study^21^. The present study aims to provide an ONSSA assessment using portable ultrasonography that might be required in the space station and certain clinical scenarios. However, although 7 mm can be seen in non-portable ultrasound measurements (Philips, Bothell, Washington, USA), distinguishing the outline edge at 7 mm was hard in some of these portable ultrasound images. Thus, the ONSSA was calculated perpendicular to the vertical axis of the scanning region 3 ~ 5 mm behind the optic disc, with excellent intra- and inter-observer agreement in this study. ONSSA was employed as a surrogate for the volume of the optic subarachnoid space, which is the transversal sectional 2D area of a single ultrasound slice calculated between 3 and 5 mm posterior to the optic disc. Although it is possible to derive an estimated volume of the optic subarachnoid space from the ONSSA data, the two measures would consistently correlate and may not entirely eliminate asymmetric deformation. To further enhance predictive power, images would need to be captured in multiple planes to enable 3D modeling and ultimately achieve a more accurate volume prediction. Nevertheless, this study provides an advanced and standardized quantitative method, and ONSSA serves as a robust surrogate for monitoring optic subarachnoid space volume variations in both the weightless and clinical scenarios.

Our results partially align with most of the research indicating that microgravity can lead to increased ICP or ONS distension. However, such mild elevation has little clinical significance. Mader et al. reported borderline high lumbar puncture ICP in 4 astronauts after approximately 6-month spaceflight mission^4^. They also reported ONS distension through MRI in multiple cases. However, ONS distension was primarily subjectively observed rather than quantified^2,4,37^. Only two cases have reported quantified ONSD. The first case involved an astronaut who underwent two long-duration space flights (6 months each, 9 years apart). During the first flight, ONSD measurements were not conducted, but during the second flight, ONSD increased from ONSD range of 6.2 ~ 7 mm on pre-flight ultrasound and MRI to 6.8 ~ 7.4 mm on inflight ultrasound. These changes persisted in post-flight ultrasound and MRI assessments (0.73–0.75 mm)^6^. The other case showed asymmetric pre-flight ONSD (9.48 mm right and 6.00 mm left), and they increased following long-duration space flight (9.93 mm right and 8.30 mm left), which returned to pre-flight values 7 years post-flight^3^. Additionally, Kramer et al. reported ONSD distention (mean, 6.2 ± 1.1 mm) in 27 astronauts with MRI after space flight^11^. Sirek et al. assessed ONSD by ultrasound at 3 ~ 4 mm posterior to the optic disc in 13 astronauts and found an average increase of 0.91 mm or about 11% from pre-flight to in-flight, which remained distended post-flight^10^. ONSD at 30° and 6° HDT for 20 minutes increased by 0.5 mm and 0.031 mm relative to supine values^10^. This study also found that, in comparison to R + 180, ONSD increased by 0.15 ~ 0.21 mm at 3 mm and 0.28 ~ 0.29 mm at 5 mm; ONSSW increased by 0.21 ~ 0.27 mm at 3 mm and 0.24 ~ 0.31 mm at 5 mm; ONSSA increased by 0.44 ~ 0.46 mm^2^. Although these changes just approach the theoretical lateral resolution limits of ultrasound probes (0.4 mm), they were consistent with previously reported HDT changes measured by MRI or ultrasound^6,10,38,39^. While these mild variations may have limited clinical significance^24^, their potential effects on eye structure and function warrant future investigation. The absence of baseline data also limited the interpretation of these subtle differences. As ONSD measurements are close to detection level values, Stephanie evaluated the potential of using Δ (ONSD – OND) rather than ONSD. Despite the limitations posed by their small study sample, a correlation of ICP and Δ (ONSD – OND) with r = 0.65 and an ROC analysis with an AUC of 0.79 were satisfactory in their population, suggesting that Δ (ONSD – OND) might provide a more reliable estimation of ICP than ONSD^40^. Consequently, we introduced ONSSW as Δ (ONSDint – OND) to further demonstrate the ONS deformation due to long-term microgravity. The ONS parameters consistently exhibited statistically significant distension, except for the ONSD 3 mm. This may be due to the relatively small sample, as we only assessed optic sheath distension in a single point, which was less sensitive to reflect optic nerve subarachnoid space volume variation. Furthermore, the R + 180 anatomic measurements may have been altered somewhat by the previous period of HDT and thus decrease the magnitude of variation. The difference in ONSD changes at 5 mm and 3 mm might be arised from the inhomogeneity in the collagen fibers of the ONS and the uneven stress distribution across the optic sheath wall. Some studies have found comparable ONSD after space flight missions or HDT BR. For instance, Rhor et al. reported a comparable ONS cross-sectional area at 3 mm posterior to the optic disc using MRI in 10 astronauts immediately after a 6-month flight^36^. However, a subject diagnosed with optic disc edema displayed an increased ONS cross-sectional area post-flight. Compared with MRI, ultrasonography is easy-to-perform, portable, rapid, and reproducible to perform in space flight. NASA’s 70-day HDT study did not report ONSD and ICP results, but it revealed subtle ocular changes, suggesting that HDT might have little effect on ICP^41,42^. Lawley et al. proposed that the pillows placed under HDT subjects’ heads might provide a sufficient gravitational vector for blood and CSF to drain from the head and prevent ICP elevation. They suggest that ICP in supine subjects were reduced by an average of 4 mmHg when the head was elevated onto a pillow^43^.

To overcome the potential influence of body position and circadian rhythms on ONSP, all tests in this study were performed by a portable ultrasound device beside the bed in the morning. Additionally, volunteers were examined in a supine position without pillows. The study sample was larger than previous studies, with repeated measurements over multiple time points. We observed an increased head circumference and decreased hipline among participants, which may indicate a microgravity-induced cephalad fluids shift. HDT might increase peri-optic cerebrospinal fluid hydrodynamics and affect cerebrospinal fluid volume and movement within the optic nerve subarachnoid space^44^. Moreover, HDT might increase MBP and thus potentially contribute to the increase of cerebrospinal fluid volume, which could explain the association between changes in MBP and ONSSA^45^. ICP is influenced by various factors, including venous pressures, secretion rate, and frictional resistance to fluid drainage. CSF is primarily produced by the choroid plexuses and circulates from the ventricles into basal cisterns. A complex autoregulation system maintains the dynamic of CSF and the homeostasis of ICP. MBP is positively correlated to ICP in normal subjects^5^. The elevated MBP induced by HDT may increase CSF production from choroid plexuses, thereby contributing to heightened ICP. Studies have shown that steady-state CSF and dynamic cerebral autoregulation can be preserved during short-term -30° HDT despite the higher ICP compared to -10° HDT^46^. However, the preservation of such autoregulation during prolonged HDT deserves further study. Additionally, the hypothesis of HDT-induced compartmentalization of cerebrospinal fluid in the optic nerve subarachnoid space with locally elevated ONSP has been proposed as another explanation for ONSSA distention^1,3^.

**Fig. 4:**
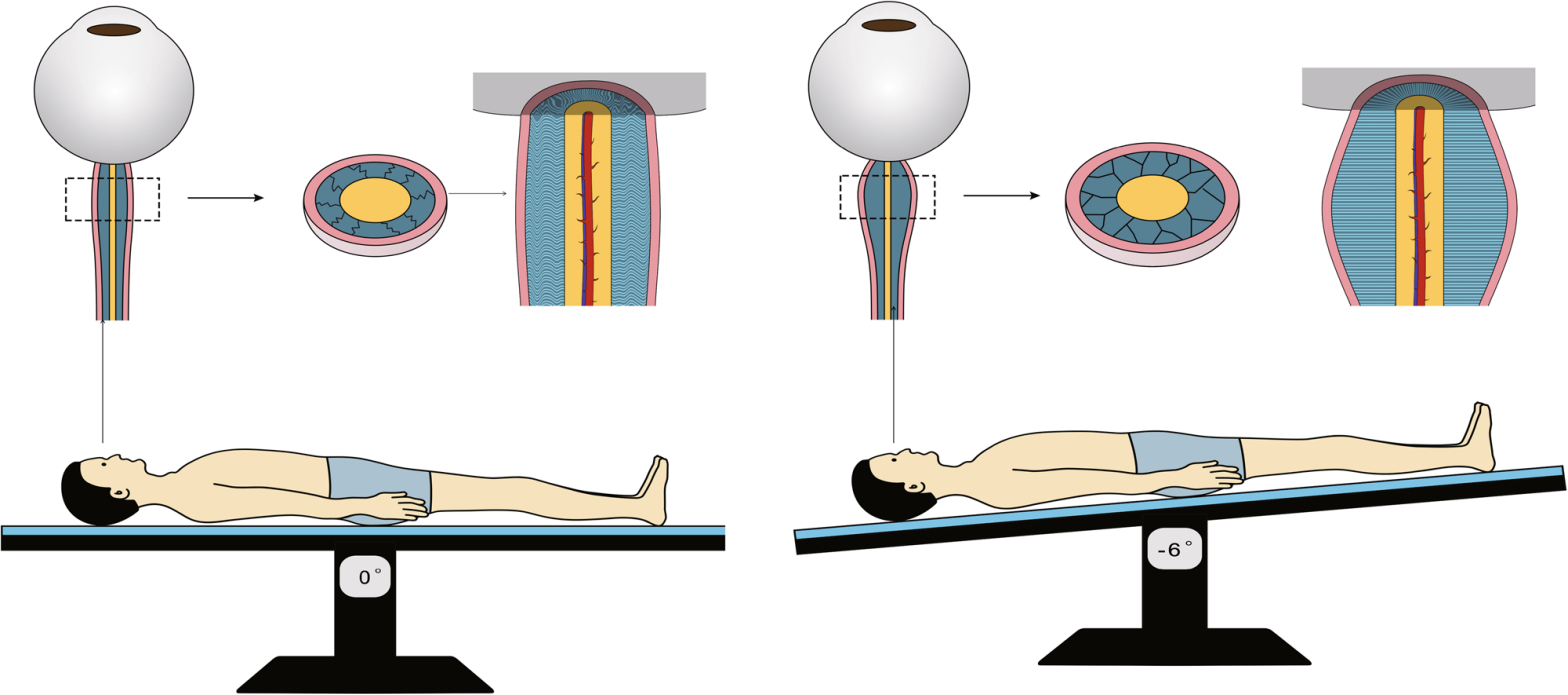
Hypothesized schematic of the microgravity-induced optic subarachnoid space distension. The presumably folded trabeculae were likely stretched, and the optic nerve subarachnoid space was expanded during the head-down tilt bed rest.

The distension of the ONSSA or the immediate rise in ICP during HDT has been demonstrated in human subjects and animal models^47,48^. Gradual return toward the pre-HDT baseline has been noted in several studies^39,48,49^. Permanent changes after long-duration spaceflight have also been observed^3,4,6,50^. In the present study, due to the absence of the pre-HDT baseline and the first 30 days of HDT data, we only observed consistent ONSSA distension during HDT compared to the supine position on the 180-day post-HDT. Gradual increase in the expansion or gradual return toward the pre-HDT baseline was not observed. We speculate that this may be due to two reasons: first, the gradual expansion period may have occurred within 30 days of HDT and reached a plateau afterward; second, the increase in ONSP during HDT might not follow a process of gradual increase over time. Alternatively, it may occur after a long adaptation period, with a subsequent gradual recovery of ONSP, which we did not capture in this study. The trabeculae of the optic nerve subarachnoid space may be initially folded and collapsed. As a result, any increase in pressure would stretch the trabeculae and open up a reserve space^51^. The increased porosity of trabecular fibers immediately after an ICP increase (leading to ONS dilation) remains 30 days post-procedure, even though the size of the ONSD returns to near-normal levels^20^. Moreover, the optic nerve sheath may possess exceptional elasticity properties, allowing considerable distension^34^. The immediate retrobulbar space is more distensible due to the absence of pillars and septae found in the less distensible intraorbital and canalicular portions of the ONS^7,34^. As illustrated in Fig. 4, the presumably folded trabeculae were likely stretched, and the optic nerve subarachnoid space was expanded during HDT. Additionally, there is a cross-over point where the distention of ONS ceases, regardless of the increasing ICP^9^. Plastic deformation is likely to occur at ICP greater than 45 mmHg. However, no studies are currently exploring the time-dependent viscoelastic properties of the ONSD to determine the potential for hysteresis in ONSD measurements^24^. Our findings indicate a mild yet consistent ONSSA distension across three-time points during HDT, followed by an immediate decrease after HDT, suggesting that ONSSA during the 90-day HDT might not have reached a cross-over point or exceeded the pressure or time threshold for plastic deformation. However, our study cannot answer whether ONSP on R + 180 recovered to pre-HDT levels, which warrants further investigation.

While increased ONSP can lead to axoplasmic flow stasis and optic disc edema, no OND distension was observed in this study. It is suggested that ONS remodeling would offset the impact of ONSP on axoplasmic flow stasis unless the pressure exceeds a cut-off value. The cut-off values for ONSD at 3 mm posterior to the global were found to be 5.8 ~ 6.4 mm^12^. The ONSD in the present study was below 5.8 mm, which helps explain the consistent OND observed.

The limitations of this study include mainly four aspects. Firstly, this study included a relatively young cohort compared to the average age of astronauts experiencing SANS (about 25 to 50 years old), and age may be a factor in SANS due to decreased mechanical compliance of structures with the increase of age, including the optic sheath. Secondly, this study only included male subjects. Hence, it remains unclear whether similar ONSSA changes would be observed in females due to gender differences. Thirdly, similar to the measurement of the ONSD, ONSSA was hand-drawn and the accuracy of ONSSA measurement depends on the performance of the operator, and the need for specialized training to perform and interpret the ultrasound tests. Therefore, the development of automated methods like artificial intelligence analysis for measuring ONS geometries could enhance the accuracy. Finally, this study did not record the pre-HDT values of the optic nerve sheath parameters, which makes it challenging to determine whether ONSSA had recovered to pre-HDT on R + 180. However, the primary goal of this study was to develop a reliable measurement method using ultrasound techniques to study the effects of microgravity on surrogate measurements of ONSP. The results of this study confirmed the presence of ONSSA distension during long-term HDT when compared to the supine values.

Briefly, this study effectively avoided several limitations reported in existing ONSD literature and found that the ONSSA expansion during HDT and immediately recovered after HDT on R + 2 in comparison to the R + 180 level. The results further confirmed that microgravity-induced ONS plastic changes may be due to the elevated ONSP. However, these changes were relatively small and of little clinical significance. The use of portable ultrasound-based ONS assessment, particularly the 2D transversal sectional area of the optic subarachnoid space between 3 mm and 5 mm posterior to the optic disc vitreoretinal interface, may constitute a noninvasive, cost-effective, and highly portable dynamic approach for monitoring ONS deformation during spaceflight and in terrestrial clinical settings. Whether chronic and mild ONSSA distension has adverse effects on eye structure and function during prolonged HDT and space flight deserves further study.

## Methods

### Study design

The present 90-day strict 6°HDT BR cohort study was conducted at the Space Institute of Southern China (SISC, Shenzhen, China) in 2019. The study protocol was approved by the ethics committee of the Astronaut Center of China (registration number ACC201904). The study protocol adhered to the tenets of the Declaration of Helsinki. Prior to the experiment, written informed consent was obtained from all participants after an explanation of the experimental procedures and associated risks. All participants signed the consent form. This study included a total of 36 Chinese nonathletic male subjects who underwent extensive physical and psychological examinations. The exclusion criteria were: (1) presence of any eye diseases; (2) myopic refractive error exceeding eight diopters; (3) diagnosis of neurological, cardiovascular, mental, or infectious diseases; (4) incompatibility to bed rest studies (inability to tolerate 90 days of confinement to bed rest, close spaces that would preclude MRI testing, or sharing a hospital room with another subject). During the study, all participants strictly maintained a 6°HDT position without using a pillow for a continuous 90-day period, with constant monitoring via 24-hour camera surveillance. They were transported to measurement sites using a 6 ° tilt transfer bed by professional nurses. Measurements were performed by professional doctors on HDT day 30 (HDT30), 60 (HDT 60), 90 (HDT 90), recovery day 2 (R + 2), 7 (R + 7), 14 (R + 14), 30 (R + 30), 90 (R + 90), and 180 (R + 180). From pre-HDT to R + 30, the participants adhered to a controlled daily diet and routine. After one month of recovery, the participants were discharged from the research unit and returned on R + 90 and R + 180 for follow-up measurements. Systolic blood pressure (SBP), diastolic blood pressure (DBP), and pulse rate (PR) were measured using an electronic sphygmomanometer (HEM-7133, Omron Corporation, Kyoto) on the right brachial artery at heart-level height with the subjects in a supine position after at least a 5-minute period without physical activity. The mean arterial blood pressure (MBP) was determined by the equation: MBP = 1/3 × SBP + 2/3 × DBP^52^. The Body Mass Index (BMI) was calculated as weight/height^2^. The head circumference was measured around the skull and across the glabella and occipital bone^53,54^. The waist circumference was measured at the midway between the lowest rib and the iliac crest, and the hip circumference was measured at the level of the great trochanters were measured with flexible inelastic tape^55^. These three measurements were assessed in duplicate and averaged for further statistical analysis.

### Trans-orbital ultrasonography imaging

For bedside convenience, this study employed a portable ultrasound machine (X5 model from SonoScape®, Shenzhen, China). The ocular ultrasound images from both eyes of all participants were acquired using a 12 MHz linear array probe (L741; SonoScape®, Shenzhen, China) in a 6° HDT and supine position during the 90-day BR and in the supine position during the recovery in the morning by an investigator (FYD) who is experienced in the use of trans-orbital ultrasonography in a masked manner. The study admitted the “As Low As Reasonable Achievable” (ALARA) principle and FDA-recommended MI index, which was lower than 0.23. During the examination, the eye under assessment was kept closed, while the fellow eye focused on a fixation target above. After applying the coupling gel, the transducer was placed horizontally over the upper eyelid of the eye under examination. This sonographic section provided a transverse view of the globe and the retrobulbar structures (Fig. 3A). Images were captured when the optic nerve subarachnoid space width was maximal, and these images were stored for subsequent offline analysis. Afterward, these images were evaluated by two experienced observers (FYD and SYQ) in a masked manner using ImageJ (http://rsbweb.nih.gov/ij/).

### Image analysis

ONS parameters, including OND, ONSD, ONSSW, and ONSSA, were measured binocularly, as illustrated in Fig. 3. The vitreoretinal interface of the optic disc was defined as the label of the depth marker. As Fig. 3B and C illustrated, the pia mater appeared as a dark structure fused with the optic nerve (also dark), while the subarachnoid space was characterized by two hyperechoic striped bands. The dura mater was shown as a dark line between the white retrobulbar fat and the hyperechoic striped bands. OND and ONSD were measured at 3 mm and 5 mm from the optic disc surface (vitreoretinal interface), and OND was marked by the hyperechoic inner boundary on both sides (S1), while ONSD was marked by the hyperechoic outer boundary on both sides (S2). The ONSSW at these specific locations was calculated as ONSD minus OND. The entire ONSSA between 3 to 5 mm posterior to the optic disc was outlined using a “Freehand selections” tool. After tracing, the ImageJ software (National Institutes of Health, USA, version: 1.52a) automatically calculated the ONSSA. For each ultrasound image, measurements were calibrated using the scale at the base of the image in the ultrasound system and were conducted 3 times to get the average value. Inter and intra-observer reliability were calculated. To calculate intra-observer reliability, 100 images were randomly selected and repeated after one month.

### Statistical analysis

The normal distribution was assessed using the Kolmogorov-Smirnov test. Descriptive statistics for all the parameters were presented as means ± SD. The mixed linear model was used to compare repeated measurements of ONS parameters at each time point to adjust for the correlation between the two eyes of each subject. Repeated-measures ANOVA was applied to analyze the differences among BMI, blood pressure, waist, hip, and head circumference at exam time points. Multiple comparisons were adjusted using Bonferroni correction. Univariate linear regression was applied to identify the association between changes in ONSSA and other systemic parameters during the HDT BR period compared to R + 180. Inter- and intra-observer reliability was determined using the intraclass correlation coefficient (ICC). A statistical significance level of *P* < 0.05 was considered. All statistical analyses were conducted using commercially available statistical software packages (SPSS for Mac, v. 24.0, IBM-SPSS, Chicago, IL).

### Reporting summary

Further information on research design is available in the Nature Research Reporting Summary linked to this article.

### Supplementary information

Supplementary figure 1- Representative images of optic nerve subarachnoid space area between 3 and 5mm posterior to the optic disc.pdf

Reporting Summary

## Data Availability

The datasets (ultrasonography image in JPG. format) generated and analyzed during this study are not publicly accessible due to containing personal details of subjects but can be available upon reasonable request to the corresponding authors.
