# Supplementary material for: Quantitative ultrasound image assessment of the optic nerve subarachnoid space during 90-day head-down tilt bed rest
Source: NPJ Microgravity. 2024 Jan 17;10:9. doi: 10.1038/s41526-024-00347-x (PMC10794463; doi:10.1038/s41526-024-00347-x)
Supplement: Supplementary file 1 — Supplementary figure 1- Representative images of optic nerve subarachnoid space area between 3 and 5mm posterior to the optic disc.pdf [file 41526_2024_347_MOESM1_ESM.pdf]

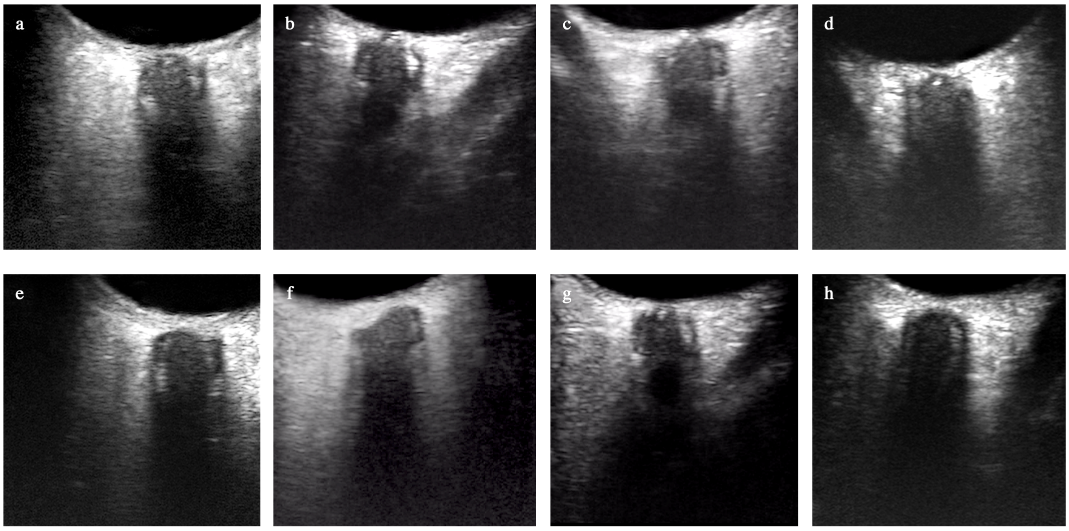

Supplementary Figure 1.Representative images of optic nerve subarachnoid space area between 3 and 5mm posterior to the optic disc (unmarked) variations during the 90-day head down tilt bed rest and on recovery day 180. ONSSA distension were showed on HDT30d(a,e), HDT 60d(b,f), HDT 90d(c,g), compared with R+180d(d,h). The top and bottom lines indicated ONSSA in the right and left eye, respectively. HDT =head down tilt bed rest; R=recovery time.
