# Supplementary material for: Quantitative ultrasound image assessment of the optic nerve subarachnoid space during 90-day head-down tilt bed rest
Source: NPJ Microgravity. 2024 Jan 17;10:9. doi: 10.1038/s41526-024-00347-x (PMC10794463; doi:10.1038/s41526-024-00347-x)
Supplement: Supplementary file 2 — Reporting Summary [file 41526_2024_347_MOESM2_ESM.pdf]

nature portfolio

Corresponding author(s): Ningli Wang , Yinghui Li

Last updated by author(s): Oct 10, 2023

Reporting Summary

Nature Portfolio wishes to improve the reproducibility of the work that we publish. This form provides structure for consistency and transparency in reporting. For further information on Nature Portfolio policies, see our Editorial Policies and the Editorial Policy Checklist.

Statistics

For all statistical analyses, confirm that the following items are present in the figure legend, table legend, main text, or Methods section.

n/a	Confirmed	
☐	☒	The exact sample size (*n*) for each experimental group/condition, given as a discrete number and unit of measurement
☐	☒	A statement on whether measurements were taken from distinct samples or whether the same sample was measured repeatedly
☐	☒	The statistical test(s) used AND whether they are one- or two-sided<br>*Only common tests should be described solely by name; describe more complex techniques in the Methods section.*
☐	☒	A description of all covariates tested
☐	☒	A description of any assumptions or corrections, such as tests of normality and adjustment for multiple comparisons
☐	☒	A full description of the statistical parameters including central tendency (e.g. means) or other basic estimates (e.g. regression coefficient) AND variation (e.g. standard deviation) or associated estimates of uncertainty (e.g. confidence intervals)
☐	☒	For null hypothesis testing, the test statistic (e.g. *F*, *t*, *r*) with confidence intervals, effect sizes, degrees of freedom and *P* value noted<br>*Give P values as exact values whenever suitable.*
☒	☐	For Bayesian analysis, information on the choice of priors and Markov chain Monte Carlo settings
☒	☐	For hierarchical and complex designs, identification of the appropriate level for tests and full reporting of outcomes
☒	☐	Estimates of effect sizes (e.g. Cohen's *d*, Pearson's *r*), indicating how they were calculated

Our web collection on statistics for biologists contains articles on many of the points above.

Software and code

Policy information about availability of computer code

Data collection	*Provide a description of all commercial, open source and custom code used to collect the data in this study, specifying the version used OR state that no software was used.*
Data analysis	*Provide a description of all commercial, open source and custom code used to analyse the data in this study, specifying the version used OR state that no software was used.*

For manuscripts utilizing custom algorithms or software that are central to the research but not yet described in published literature, software must be made available to editors and reviewers. We strongly encourage code deposition in a community repository (e.g. GitHub). See the Nature Portfolio guidelines for submitting code & software for further information.

Data

Policy information about availability of data

All manuscripts must include a data availability statement. This statement should provide the following information, where applicable:

- Accession codes, unique identifiers, or web links for publicly available datasets
- A description of any restrictions on data availability
- For clinical datasets or third party data, please ensure that the statement adheres to our policy

The datasets (ultrasonography image in JPG. format) generated and analyzed during this study are not publicly accessible due to containing personal details of subjects but can be available upon reasonable request to the corresponding authors.

Research involving human participants, their data, or biological material

Policy information about studies with human participants or human data. See also policy information about sex, gender (identity/presentation), and sexual orientation and race, ethnicity and racism.

Reporting on sex and gender	This study included a total of 36 Chinese nonathletic male subjects.Hence, it remains unclear whether similar ONSSA changes would be observed in females due to gender differences.
Reporting on race, ethnicity, or other socially relevant groupings	This study included a total of 36 Chinese subjects.
Population characteristics	This longitudinal study included 72 eyes from 36 Chinese male volunteers, who had a mean (SD) age of 32.4 (5) years and a mean (SD) body mass index (BMI) of 22.20 (2.12) kg/m2 before HDT. The exclusion criteria were: (1) presence of any eye diseases; (2) myopic refractive error exceeding eight diopters; (3) diagnosis of neurological, cardiovascular, mental, or infectious diseases; (4) incompatibility to bed rest studies (inability to tolerate 90 days of confinement to bed rest, close spaces that would preclude MRI testing, or sharing a hospital room with another subject). All the subjects completed a strict 90-day HDT BR. Because the pre-HDT ONS data were not collected, the baseline level was established using the R+180 data.
Recruitment	This study included a total of 36 Chinese nonathletic male subjects through advertisement who underwent extensive physical and psychological examinations.
Ethics oversight	The study protocol was approved by the ethics committee of the Astronaut Center of China (registration number ACC201904). The study protocol adhered to the tenets of the Declaration of Helsinki. Prior to the experiment, written informed consent was obtained from all participants after an explanation of the experimental procedures and associated risks. All participants signed the consent form.

Note that full information on the approval of the study protocol must also be provided in the manuscript.

Field-specific reporting

Please select the one below that is the best fit for your research. If you are not sure, read the appropriate sections before making your selection.

☒ Life sciences ☐ Behavioural & social sciences ☐ Ecological, evolutionary & environmental sciences

For a reference copy of the document with all sections, see nature.com/documents/nr-reporting-summary-flat.pdf

Life sciences study design

All studies must disclose on these points even when the disclosure is negative.

Sample size	NASA 70-day Bed Rest study included 29 subjects. According to 20% dropout rate, 36 subjects were included in this study infally.
Data exclusions	No data were excluded from the analysis.
Replication	For each ultrasound image, measurements were conducted 3 times to get the average value. Inter and intra-observer reliability were calculated. To calculate intra-observer reliability, 100 images were randomly selected and repeated after one month.
Randomization	This is not relevant to this study.
Blinding	These ultrasound images were evaluated by two experienced observers (FYD and SYQ) in a masked manner using ImageJ (http://rsbweb.nih.gov/ij/).

Reporting for specific materials, systems and methods

We require information from authors about some types of materials, experimental systems and methods used in many studies. Here, indicate whether each material, system or method listed is relevant to your study. If you are not sure if a list item applies to your research, read the appropriate section before selecting a response.

Materials & experimental systems

n/a	Involved in the study
☒	☐ Antibodies
☒	☐ Eukaryotic cell lines
☒	☐ Palaeontology and archaeology
☒	☐ Animals and other organisms
☒	☐ Clinical data
☒	☐ Dual use research of concern
☒	☐ Plants

Methods

n/a	Involved in the study
☒	☐ ChIP-seq
☒	☐ Flow cytometry
☒	☐ MRI-based neuroimaging

Plants

Seed stocks	*Report on the source of all seed stocks or other plant material used. If applicable, state the seed stock centre and catalogue number. If plant specimens were collected from the field, describe the collection location, date and sampling procedures.*
Novel plant genotypes	*Describe the methods by which all novel plant genotypes were produced. This includes those generated by transgenic approaches, gene editing, chemical/radiation-based mutagenesis and hybridization. For transgenic lines, describe the transformation method, the number of independent lines analyzed and the generation upon which experiments were performed. For gene-edited lines, describe the editor used, the endogenous sequence targeted for editing, the targeting guide RNA sequence (if applicable) and how the editor was applied.*
Authentication	*Describe any authentication procedures for each seed stock used or novel genotype generated. Describe any experiments used to assess the effect of a mutation and, where applicable, how potential secondary effects (e.g. second site T-DNA insertions, mosiacism, off-target gene editing) were examined.*
